# Reversing the Luminance Polarity of Control Faces: Why Are Some Negative Faces Harder to Recognize, but Easier to See?

**DOI:** 10.3389/fpsyg.2020.609045

**Published:** 2021-01-21

**Authors:** Abigail L. M. Webb

**Affiliations:** Department of Psychology, University of Essex, Colchester, United Kingdom

**Keywords:** face perception, control faces, luminance polarity, skewness, facial recognition, apparent contrast, negative faces, spatial inversion

## Abstract

Control stimuli are key for understanding the extent to which face processing relies on holistic processing, and affective evaluation versus the encoding of low-level image properties. Luminance polarity (LP) reversal combined with face inversion is a popular tool for severely disrupting the recognition of face controls. However, recent findings demonstrate visibility-recognition trade-offs for LP-reversed faces, where these face controls sometimes appear more salient despite being harder to recognize. The present report brings together findings from image analysis, simple stimuli, and behavioral data for facial recognition and visibility, in an attempt to disentangle instances where LP-reversed control faces are associated with a performance bias in terms of their perceived salience. These findings have important implications for studies of subjective face appearance, and highlight that future research must be aware of behavioral artifacts due to the possibility of trade-off effects.

## Introduction

### Using the Face Inversion Effect to Create Face Controls

The human visual system is especially good at interpreting information from the faces of others (Farah et al., 1995; Yovel and Kanwisher, 2004; Eimer and Holmes, 2007). It is generally accepted that this is due to the evolution of specialized and dedicated cognitive mechanisms for identifying and categorizing faces (Schmidt and Cohn, 2001; Lewis and Edmonds, 2003). The ability to read the faces of others is robustly evidenced by the face inversion effect (FIE), where face targets that are spatially inverted (rotated by 180°) are consistently associated with impaired performance on identification and recognition tasks – a renowned phenomenon confirming that humans are experts in face perception (Ellis, 1975; Valentine and Bruce, 1986; Farah et al., 1995; Lewis and Edmonds, 2003). The FIE demonstrates the importance of upright information extraction for typical face perception as it occurs in natural viewing (Farah et al., 1995; Yovel, 2016), including the role of first- and second-order properties for configural face processing. First-order properties refer to the crude configuration of features that tend to be broadly consistent across all human faces, including top-heavy information, with eyes symmetrically located either side of the center in the top half of the face, and the mouth appearing toward the bottom, below a roughly centrally located nose (Diamond and Carey, 1986; Farah et al., 1995). Second-order facial properties, on the other hand, determine the configuration of a face according to the interdependent and often idiosyncratic spatial relationships *between* individual features. Face inversion inherently disrupts the first-order relationships within a face, but maintains information regarding the spatial relationships between face parts, as under inversion these remain the same (Farah et al., 1995; Yovel, 2016). When first-order relational information is disrupted by inversion, facial recognition is markedly impaired for face targets as a consequence (Yovel, 2016).

The FIE is a valuable tool for exploring the kind of information required for successful facial recognition because it demonstrates the extent that performance on a given task is impaired rather than preserved when first-order relational information is disrupted in inverted face controls. If performance on a given task remains the same for both upright *and* inverted faces, it is typically concluded that the mechanisms for successful performance operate independently of such configural, or holistic processing. In psychophysical studies, inverted control faces are also key tools for exploring the degree to which aspects of face expression perception are driven by (1) configural processing, including the way in which their affective and emotional content is evaluated, versus (2) simple advantages afforded by their low-level image properties (Yang et al., 2007; Gray et al., 2013; Stein et al., 2014). This dissociation between the roles of affective content versus low-level image properties for task performance is possible because certain low-level image properties are preserved within inverted faces, despite the loss of configural content required for affective evaluation. Therefore an effect for upright faces that remains present for inverted faces suggests an effect that is modulated by image properties retained in control faces. An example from expression perception literature comes from studies of perceptual threat biases for fearful face expressions, where biases for detecting consciously suppressed, or simple localization of fear expressions, are preserved for both upright and inverted versions of fearful faces (Yang et al., 2007; Gray et al., 2013; Stein et al., 2014). Such findings suggest that biases remain operable in the absence of configural information, and thus independently of recognition.

However, the effect of inversion on recognition impairment is limited in that although performance is markedly worse for inverted compared to upright expressions, it can still remain *above* chance-level (Prkachin, 2003; Itier and Taylor, 2004; Russell et al., 2006; Gray et al., 2013). But when inversion and luminance polarity (LP) reversal are used together, they have an “additive” detrimental effect on facial recognition, often reported as greater than when either manipulation is used alone (Itier and Taylor, 2004; Russell et al., 2006; Gray et al., 2013). Conjoined use of spatial inversion and LP reversal is also valuable for equalizing recognition differences between facial emotions that otherwise differ above chance performance for inverted-only faces. For example, expressions of inverted happy and surprise are easier to discriminate compared to anger and fearful counterparts (Prkachin, 2003), but using both LP reversal and inversion together reduces these differences to chance, where expression-related differences are less likely to leave affective biases intact (Gray et al., 2013).

### Luminance Polarity Reversal and Spatial Inversion: Additive Impairment Effects for Face Controls

Luminance polarity refers to the luminance of each pixel in the image relative to the mean luminance. The way in which individual pixel luminance intensities are dispersed between the light-dark continuum refers to an image’s skewness distribution; information relating to cues useful for object identification, including surface properties (Vuong et al., 2005; Russell et al., 2006; Haun and Peli, 2013). LP reversal “switches” individual luminance intensities, where each pixel is subtracted from the image’s maximum intensity value. In other words, the brightest points become the darkest, and vice versa. For an upright face image whose intensity distribution is mostly skewed toward brighter pixels, this distribution becomes skewed toward mostly darker pixels. The appearance of facial stimuli subjected to LP reversal resembles that of a photographic negative.

Luminance polarity reversal has been used for its additive effects on recognition when used in conjunction with spatial inversion (Galper, 1970; Itier et al., 2006; Rossion et al., 2012; Gray et al., 2013; Hedger et al., 2019), where the accuracy and time taken to identify a face is impaired more so than when either manipulation is used alone (Hole et al., 1999; Prkachin, 2003; Rossion et al., 2012; Gray et al., 2013). Their individual effects on performance are similar in magnitude, such that although relative effects on performance differ substantially from upright faces, they often do not differ between inverted-only or LP-reversed-only faces (Kemp et al., 1990; Itier and Taylor, 2004; Gray et al., 2013). Together, the two manipulations can achieve dual disruption effects on recognition performance, evidenced by longer detection times and reduced accuracy, or “hit rates” (Itier and Taylor, 2004; Rossion et al., 2012; Gray et al., 2013; Hedger et al., 2015, 2019). These additive effects are described by Itier and Taylor (2002) as imposing a “perceptual deficiency” (p. 3) at the level of encoding, and indicate the role of sub-processes for encoding configural face information. Here, inverted faces are thought to impede first-order and “gist” information extraction, while LP reversed faces primarily disrupt surface properties and pigmentation, leaving the “gist” of a face relatively intact and instead disrupting second-order information related to shading, shape and surface properties (Hayes et al., 1986; Hole et al., 1999; Davies and Hoffman, 2002; Maurer et al., 2002; Itier and Taylor, 2004; Vuong et al., 2005; Russell et al., 2006). However, there is a body of findings showing that in some cases LP reversal can have unexpected, facilitatory effects on task performance for faces, and together they raise questions regarding their exact effect on the perception of control faces, and how these effects manifest differently between experimental paradigms and tasks.

### Trade-Offs for LP-Reversed Faces: Some Faces Are Harder to Identify, but Easier to See

It remains unclear whether diminished recognition as a result of LP reversal is face-specific, or the result of a broader impairment that is true for other, non-face objects (Russell et al., 2006; Liu-Shuang et al., 2015). In any case, LP reversal is a reliable and consistent tool for reducing facial recognition to a level similar to spatial inversion (Galper, 1970; Hole et al., 1999; Maurer et al., 2002; Prkachin, 2003; Itier and Taylor, 2004; Itier et al., 2006; Russell et al., 2006; Rossion et al., 2012; Gray et al., 2013), but a small body of findings showing a performance advantage for LP-reversed faces reinforces the notion that LP reversal and inversion affect face processing in different ways. These different effects are revealed, to a degree, between different tasks, but this task effect is somewhat difficult to disentangle, primarily because the vast majority of studies consider the effect of LP reversal on face perception in terms of its effects on identification accuracy, hit rates, and false alarms. There are few investigations of effects of LP reversal on facial visibility, appearance and salience, and studies of recognition seldom report accuracy and response times together.

The value of both accuracy and response time data are evidenced by Davies and Hoffman (2002), where in a change-detection task observers indicated whether identities of faces were the same or different. Although accuracy was poorer for LP-reversed versions of faces, it did not affect identification response times (Davies and Hoffman, 2002); not what one would expect given its obvious detrimental effects on recognition performance. A small body of findings show similar instances where LP reversal can *improve* the visibility of a face control (Hole et al., 1999; Webb and Hibbard, 2020; Webb et al., 2020). Hole et al. (1999) showed that although response times to pair (match or mismatch) the identity of face halves were slower for upright compared to inverted face halves (an expected chimeric-face-effect), LP-reversed faces were associated with overall faster judgments compared to non-reversed faces, regardless of inversion. Moreover, observers were more likely to incorrectly label face halves as a match when they were upright and LP-reversed. In other words, speeded response times for LP-reversed faces were not afforded the same level of accuracy as their non-reversed counterparts. While these findings have been interpreted as evidence that inversion disrupts configural information required for identification, whereas LP reversal does not (Hole et al., 1999; Lewis and Edmonds, 2003), there has been little discussion of the effects that such an image manipulation may have on the appearance of control faces. Hole et al. (1999) hypothesize that LP reversal may accentuate highly salient areas of the face in a way that could facilitate the rapidity of decisions, but this effect of LP reversal (both for faces *and* non-face images) has not been explored further until recently (Haun and Peli, 2013; Webb and Hibbard, 2020; Webb et al., 2020). In a contrast matching task where observers adjusted the physical contrast of a target face in order to perceptually match it to a reference face, control faces subjected to both inversion and LP reversal required *less* physical contrast compared to upright non-reversed faces in order to appear perceptually matched for contrast (Webb et al., 2020). In other words, such LP-reversed faces already appear more salient in terms of their apparent contrast compared to their upright, LP-retained counterparts. These findings suggest that perceptual matching relies on different stimulus information compared to that of explicit recognition, and indicate trade-off effects associated with LP reversal similar to those evidenced by Hole et al. (1999), where LP-reversed faces benefit from enhanced perceived salience while at the same time are unrecognizable (Gray et al., 2013; Webb et al., 2020).

A visibility advantage for LP-reversed faces is also sometimes observed under intraocular suppression conditions. In the breaking continuous flash suppression paradigm (b. CFS), a target face presented to one eye competes against a dynamic noise mask presented to the other eye in a bid to become the conscious percept (Gray et al., 2013; Stein et al., 2014). Response times to detect a consciously suppressed face under b. CFS are thought to index stimulus visibility, and the degree to which it is prioritized during processing. Recent findings show a detection advantage for inverted-LP-reversed faces compared to upright counterparts (Webb and Hibbard, 2020) under b. CFS, suggesting that although recognition of such faces is severely disrupted (Hole et al., 1999; Maurer et al., 2002; Prkachin, 2003; Itier and Taylor, 2004; Russell et al., 2006; Rossion et al., 2012; Gray et al., 2013), their stimulus strength may still provide an advantage against suppressing masks. Similarly, a trend for detecting LP-reversed over LP-retained faces was observed in a visual probe design, where facial stimuli are masked from awareness by noise stimuli (Hedger et al., 2019). It is important to note here, however, that detectability of LP-reversed faces was not found by Liu-Shuang et al. (2015), and the presence of these effects under b. CFS is mixed, where sometimes normal faces are detected faster compared to inverted-LP-reversed counterparts (Gray et al., 2013; Stein et al., 2018; Hedger et al., 2019). These contrasting results suggest possible interactions between masks and backgrounds against which stimuli are presented (effects discussed in the next paragraph). Indeed, the role of b. CFS masks on variable stimulus visibility is in itself an ongoing debate (Yang et al., 2014; Zhu et al., 2016; Webb and Hibbard, 2020).

The above findings also evidence instances where expression-related differences for face visibility may be emphasized by LP-reversal, in the form of a bias favouring the detection of emotionally-negative facial expressions. Here, visual attentional biases are observed for LP-reversed emotional faces compared to neutral faces, where this emotion bias effect is smaller for LP-retained versions of the same faces (Hedger et al., 2019). Mixed findings for expression effects are again observed for b. CFS data. For example, in one instance detection biases for different facial expressions varied according to spatial frequency and contrast, but importantly, revealed expression differences unique to LP-reversed control conditions (Webb and Hibbard, 2020). On the other hand, other b. CFS data show that while expression-related differences remain consistent between LP-reversed, inverted, and upright faces, the *magnitude* of these differences are larger for LP-reversed versus LP-retained faces (Gray et al., 2013). It is unclear why these differences occur between two localization tasks that are so similar in their procedures. Finally, in terms of the subjective and explicit appearance of expressions, LP reversal improves the *overall* perceived, or apparent contrast of facial stimuli, and *reduces* the apparent contrast advantage found for regular (retained-LP) fearful faces, but *increases* this advantage when filtered to contain high spatial frequency information (Webb et al., 2020). There therefore seems to be a complex interaction between the effects of LP reversal on the recognition and visibility of facial expressions, and the low-level image properties they are composed of. The mixed effects of LP reversal for different facial expressions do not appear to be attributable to the task alone.

Together, these findings show that LP reversal has the potential to increase the visibility and perceived salience of target faces, but importantly, they also demonstrate the need for a better understanding of how such effects are influenced by the task at hand, the effect of facial expression, and the possibility of inadvertently introducing recognition-visibility trade-off effects in behavioral data. This is discussed in further detail below.

### How Might LP Reversal Increase Faces’ Salience, or Visibility?

An explanation for the visibility effects associated with LP reversal comes from Haun and Peli (2013). In a contrast evaluation task where observers selected one of two images for the highest contrast, judgments of brightness were biased for images containing more darker compared to lighter regions. The authors propose that the subjective salience of an image, measured by its apparent contrast, is significantly influenced by local dark regions contained within it. Indeed, this is consistent with studies of simpler stimuli, including basic patches of fixed luminance values. For example, the visual system is particularly sensitive to changes in brightness for stimuli with negative (dark) compared to positive (bright) luminance, where discerning changes in luminance are more concentrated for negative than positive stimuli (Kane and Bertalmiío, 2016). Haun and Peli (2013) propose that this dark bias could be explained by the density of dark-sensitive contrast-encoding neurons at primary visual areas, where such physiological factors could influence perceptual biases, or gains, for images containing more darker regions when forming judgments of apparent contrast (Haun and Peli, 2013). Elements of an image eliciting dark biases may explain why LP reversed faces, containing more negative than bright regions, appear more salient in terms of visibility and detection thresholds (Hole et al., 1999; Webb and Hibbard, 2020; Webb et al., 2020). This notion is also supported by the “crispening” effect, where observers adjustments are more concentrated between negative patch stimuli below the intensity of a uniform background compared to those above it, suggesting, similarly to Haun and Peli (2013), that there may be greater perceptual gain for detecting brightness changes when such judgments are for largely negative as opposed to positive regions (Whittle, 1986, 1992). This may in part explain why findings of increased detectability for LP-reversed faces are inconsistently found between masking studies and those using uniform gray versus phase scrambling for backgrounds (Gray et al., 2013; Liu-Shuang et al., 2015; Stein et al., 2018; Hedger et al., 2019; Webb and Hibbard, 2020).

Because apparent contrast correlates with an image’s real-life image contrast (Haun and Peli, 2013), it is important to understand how image manipulations such as LP reversal may inadvertently change a face image’s subjective appearance. Although spatial inversion preserves image features known to influence image salience, including luminance and its distribution, contrast and spatial frequency, LP reversal does not assure the same degree of consistency. Specifically, the objective of LP reversal is to switch the brightness of pixel values, such that the distribution of light versus dark pixels is changed in the manipulated image, and so its mean luminance is inherently altered. A control face containing more dark than light pixels than its upright-self, will therefore have a *lower* average luminance. By extension of findings from Haun and Peli (2013) and the “crispening” effect, it is possible that a face control subjected to LP reversal may benefit from a boost in salience in terms of apparent contrast compared to its upright-self, simply because it contains a higher density of negative pixels in local areas. This account could explain where findings LP-reversed faces require less physical contrast in order to be perceptually matched (Webb et al., 2020) and break suppression faster compared to their upright counterparts, despite also being inverted (Webb and Hibbard, 2020). They do not, however, explain why these effects are not robustly found (Gray et al., 2013; Liu-Shuang et al., 2015; Stein et al., 2018).

Findings therefore suggest that LP reversal adjusts the physical composition of a face in a way that increases the distribution of dark pixels. *A priori*, this in turn could inadvertently increase the apparent contrast of LP-reversed faces, in a way that enhances their perceived salience. This account is upheld by some behavioral evidence from studies of perceived image salience, response times for matching judgments, and time taken to emerge from intraocular suppression (Hole et al., 1999; Webb and Hibbard, 2020; Webb et al., 2020), despite known disadvantages for recognizing such faces. As noted above, however, there are very few findings that explore LP reversal effects on face processing in terms of readiness to detect, or the visibility of such control faces. Findings that do exist show inconsistencies, though it is not clear to what extent this variability is related to task and stimulus presentation differences. Evidence thus far emphasizes the need for further research to construct a clearer understanding of the way in which LP reversal influences the perceived appearance of faces.

## Discussion

### Implications for Present and Future Research

There are two key implications of using LP reversal to create control faces. The first relates to visibility confounds that could be inadvertently introduced to facial stimuli by reversing their LP (Haun and Peli, 2013; Webb and Hibbard, 2020; Webb et al., 2020). In particular, findings, although few, thus far suggest that LP reversal has the potential to improve stimulus salience under some experimental conditions, and that a degree of this inconsistency could be task-related. In particular, visibility advantages associated with LP reversal are upheld by both behavioral findings and theoretical accounts. Here, the finding that LP-reversed control faces appear more salient in terms of their apparent contrast compared to regular (LP-retained) faces is upheld by the notion that the visual system is more informed by local dark regions as opposed to “light” areas when determining brightness (Whittle, 1986, 1992; Haun and Peli, 2013; Webb et al., 2020). Taken together, these findings imply that LP reversal has an explicit and “conscious” influence on the subjective appearance of a face. But this account cannot explain why LP reversal boosts the detectability of faces in some b. CFS studies (Webb and Hibbard, 2020), but not others (Gray et al., 2013; Stein et al., 2018; Hedger et al., 2019). Indeed, if LP reversal facilitates subjective ratings of salience, this effect should be diminished for a b. CFS task that relies on crude localization regardless of the content of faces. Despite this, LP-induced performance improvement in b. CFS is only observed in one instance (Webb and Hibbard, 2020). The notion that LP reversal effects are exclusive to subjective ratings of appearance is also not upheld by findings where response times to match the identities of faces are faster for LP-reversed faces despite identity (and therefore accuracy) impairment (Hole et al., 1999). It is, however, clear from these findings that LP reversal, like inversion, robustly impairs facial recognition. This much is clear. It is therefore likely that LP reversal alters the subjective appearance of a face as a physical stimulus, as opposed to its salience in terms of registering its expression, or extrapolating its identity. For studies concerned with the subjective *appearance* of facial expressions (or indeed non-emotional faces), it is therefore important to be mindful of visibility confounds associated with LP-reversed control faces. Particularly because in some cases, an advantage for detecting LP-reversed control faces over upright, retained faces appears at first to contradict the robust FIE (Webb and Hibbard, 2020), and moreover, has the potential to emphasize perceptual biases for detecting control versions facial expressions (Hedger et al., 2019; Webb and Hibbard, 2020). Further investigation is required to confirm the extent of these effects. But the need for consideration is clear: we must be conscious of the possibility that LP reversal can inadvertently introduce visibility-recognition trade-off effects for face controls. In cases where image statistics are thought to play an important role for performance, inversion alone may be a more appropriate method for creating face controls, particularly as it is a reliable and well-used tool for manipulating facial recognition. It also assures a greater degree of consistency between upright faces and face controls in terms of luminance and intensity distributions, such that inverted control and upright faces do not differ in their apparent, perceived contrast. It may also be possible to better control for the effects of LP reversal on image salience, where adjusting the skewness of pixel intensities in faces images *before* LP reversal would result in a control face whose pixel intensities are less skewed. Though selectively reversing the polarity of pixel intensities around the mean does not appear to facilitate response times to detect faces (Liu-Shuang et al., 2015). Moreover, while it is generally accepted that contrast equalization ensures consistency between the physical and perceived contrast of an image (Redies et al., 2008), it is reasonable to ask whether LP-reversed stimuli equalized for luminance result in smaller visibility differences between stimuli. Note, however, that this is difficult to discern, when information regarding luminance normalization and the point during stimulus generation it was employed is not always available. Transparency on the details for creating LP-reversed faces could reveal why some control faces are associated with stark visibility effects, and others not. This does, however, raise the question of whether spatial inversion would be a more efficient method.

Secondly, some evidence so far also shows that not only does LP reversal accentuate expression-related differences in face visibility (Hedger et al., 2019), but the magnitude and direction of trade-off effects also vary according to expression and spatial frequency content of faces (Webb and Hibbard, 2020; Webb et al., 2020). This is especially important for psychophysical studies of expression perception whose objective is to isolate the role of statistical image properties. Specifically, LP reversal *only* facilitates faces’ visibility when they are composed of intact broadband or low spatial frequency information, and for mid-range and high frequency versions of the same faces this visibility advantage diminishes (Webb et al., 2020). This is likely due to the loss of contrast at higher spatial frequency ranges, such that LP reversal effects on intensity distributions are lesser by comparison. Moreover, while broadband and low frequency fear expressions show a visibility advantage over other facial expressions, the reverse is true when the same faces are mid-range and high frequency filtered (Webb et al., 2020). This is likely due to natural expression-related differences between faces image composition (Menzel et al., 2018; Webb et al., 2020). While the interaction between LP reversal and spatial frequency filtering has been explored for its effect on facial recognition, the same is not true for their combined effects on face *visibility*. For example, it is thought that LP-reversal does not impair facial recognition in high-pass filtered faces because in such faces, second-order featural information is preserved by high frequency bands (Maurer et al., 2002), but these findings do not explain why detection is improved for low- rather than high-pass filtered faces (Webb and Hibbard, 2020). Finally, expression-related differences in perceptual biases for faces are also slightly emphasized for LP-reversed versions of faces compared to their normal counterparts (Hedger et al., 2019). Therefore, even for studies concerned exclusively with intact broadband faces, LP reversal, like inversion, also has irregular effects on performance for different expressions, and their appearance compared to normal counterparts (McKelvie, 1995; Prkachin, 2003; Hedger et al., 2019; Webb et al., 2020).

### Direction for Future Research

There two broad avenues for further investigation. The first is to develop the current understanding of LP reversal effects on face *visibility* as opposed to explicit *recognition*. The second is to develop a better understanding of the mechanisms that enable visibility-recognition trade-offs for facial stimuli.

Understanding the role of task-dependence is motivated by mixed outcomes of different task types, and methods for presenting face stimuli. For example, whether detection thresholds indicate awareness of gradually emerging individual face parts (Liu-Shuang et al., 2015), fully presented face parts at suprathreshold (Itier et al., 2006), with linearly increasing brightness against dynamic masks (Gray et al., 2013; Hedger et al., 2019; Webb and Hibbard, 2020), and moreover, whether the observers task is to indicate awareness of a percept in terms of its presence regardless of location (Gray et al., 2013; Hedger et al., 2015), or to provide location-specific responses (Stein et al., 2014; Webb and Hibbard, 2020). Such tasks highlight a differentiation between task type, where findings show a robust and widely reported impairment effect of LP reversal on recognition, while a smaller body of studies show suggest that LP reversal may alter the appearance of a target face in a way that promotes response time and perceived salience, while compromising accuracy. Tasks concerned with the latter are comparatively scarce and require further investigation. A greater understanding of these task-related effects is important because control faces are employed so broadly across disciplines within social and clinical sciences, and so their appropriateness and efficacy outside of psychophysical studies requires validation. Indeed, it has been suggested previously that additive and independent effects of LP reversal and inversion in themselves demonstrate that recognizing and detecting are mutually exclusive abilities that require different information (Lewis and Edmonds, 2003). It is therefore important to acknowledge and better understand the contexts where LP reversal is appropriate, including those where it can be expected to exert experimental artifacts. Currently findings regarding LP reversal and face *visibility*, as opposed to recognition, come from a small range of studies that although are behaviorally and theoretically supported (Whittle, 1986, 1992; Hole et al., 1999; Haun and Peli, 2013; Kane and Bertalmiío, 2016; Webb and Hibbard, 2020; Webb et al., 2020), require further replication.

It is also necessary to develop a more substantial understanding of both stimulus and cognitive mechanisms responsible for visibility-recognition trade-off effects for LP reversed control faces. In the first instance, such an understanding is key for the psychophysical development of stimuli. The interaction between target stimulus and background luminance, for example, may play an important role in determining the salience of the target (Whittle, 1986, 1992; Haun and Peli, 2013; Kane and Bertalmiío, 2016). Understanding this interaction is crucial for all studies that present grayscale images against uniform gray backgrounds, particularly for those interested in perceived salience. In the second instance, the additive -but not undependably different- effects associated with inversion and LP reversal for face recognition emphasize the role of sub-processes for facial recognition that are influenced differently by the two image manipulations. Further exploring manipulation-specific and combined effects on performance will provide greater insight into the cues, both low level and affective, required for optimal face recognition.

## Data Availability Statement

The original contributions presented in the study are included in the article/supplementary material, further inquiries can be directed to the corresponding author.

## Author Contributions

AW was responsible for the conceptualization of the manuscript, drafting, and completion of the final version.

## Conflict of Interest

The author declares that the research was conducted in the absence of any commercial or financial relationships that could be construed as a potential conflict of interest.
